# Attenuation of OX40 signaling suppression by age disrupts peripheral deletion of CD4^+^ T cells specific for the epidermal autoantigen desmoglein 3

**DOI:** 10.1186/s12979-023-00353-9

**Published:** 2023-06-12

**Authors:** Hisato Iriki, Miho Mukai, Yasuhiko Asahina, Yoko Kubo, Hiromi Ito, Masayuki Amagai, Hayato Takahashi

**Affiliations:** 1 Department of Dermatology, Keio University School of Medicine, 35 Shinanomachi, Shinjuku-Ku, Tokyo, 160-8582 Japan; 2 Laboratory for Skin Homeostasis, RIKEN Center for Integrative Medical Sciences, 1-7-22 Suehiro-Cho, Tsurumi-Ku, Yokohama City, Kanagawa, 230-0045 Japan

**Keywords:** Aging, Peripheral immunological tolerance, Autoimmune disease, Autoreactive T cells, Pemphigus

## Abstract

Various autoimmune responses increase with age, but the underlying mechanism is not clear. In this study, we used CD4^+^ T cells expressing a transgenic T cell receptor specific for desmoglein 3 (Dsg3), which is the target antigen of the autoimmune bullous disease pemphigus vulgaris, to examine how peripheral immunological tolerance against pathogenic autoreactive CD4^+^ T cells changes with age. Dsg3-specific T cells were deleted within 14 days after adoptive transfer into young mice (8 weeks old), while they escaped deletion when transferred into older mice over 42 weeks old. Dsg3-specific T cells produced higher levels of the proinflammatory cytokine IFN-γ in aged mice than in young mice. In addition, the expression levels of both OX40 and Birc5, which are important for cell survival in T cell clonal proliferation, were higher in aged than in young mice. The dysfunction in suppressing proinflammatory cytokine secretion and Birc5 upregulation in Dsg3-specific autoreactive T cells may reflect an aspect of the preliminary steps in autoimmune disease development in the aged population. Understanding this mechanism may lead to better risk evaluation of autoimmune disease development and to onset prevention.

**Supplementary Information:**

The online version contains supplementary material available at 10.1186/s12979-023-00353-9.

## Introduction

The immune system is regulated in a complex manner that allows it to respond to a wide variety of foreign antigens without attacking the host’s own tissues [1]. However, the occurrence of autoimmunity, in which the immune system attacks the host, increases with aging [2, 3]. In an aging world, understanding the mechanism underlying this increase is of growing importance for the development of preventative measures and treatment strategies.

Various mechanisms, referred to collectively as immune tolerance, prevent autoimmune reactions. There are two known mechanisms of preventing the occurrence of pathogenic autoreactive T cells: central tolerance, which functions during T cell maturation in the thymus; and peripheral immune tolerance, which is active in secondary lymphoid organs and peripheral tissues. In central tolerance, thymic epithelial cells express and present peripheral tissue-specific antigens as self-antigens under the control of autoimmune regulator (AIRE), Fezf2, etc., thereby eliminating T cells with T cell receptors (TCRs) that can respond to self-antigens or inducing their differentiation into regulatory T cells (Tregs) [4–7]. Peripheral tolerance has been attributed to regulatory T cells (Tregs) and tolerogenic antigen-presenting cells. Tregs secrete anti-inflammatory cytokines such as IL-10 and TGF-β, and exert their inhibitory effects on effector T cells through direct cell-to-cell contact [8–10]. Additionally, costimulatory signals provided by antigen-presenting cells, such as CD80/86, CD40, and OX40L, are crucial for T cell activation, including of pathogenic autoreactive T cells. However, the expression of these molecules on tolerogenic dendritic cells (DCs) is generally low [11–14]. Conversely, coinhibitory signals, such as PD-L1 and CTLA-4, from DCs to T cells also play a role in immune suppression [15, 16].

In the aging process, the diversity of the TCR repertoire is reduced [17], and the quality and quantity of T cells vary greatly [18]. Specifically, the Th2/Th1 ratio decreases, and the number of Tregs increases. Furthermore, IL-10 production by Tregs increases [19–21]. The age-related increase of Tregs reflects the increased resistance of aged mouse Tregs to apoptosis [22]. These changes may be involved in the age-related decline of T cell-mediated immunity against infectious diseases and tumor immunity.

Here, we focus on the involvement of aging in the risk of autoimmune disease development. Despite an increase of peripheral Treg, the diversity of antigen-specific Tregs is reduced, presumably due to a decline of newly developed Treg cells in the thymus and periphery [10, 21]. This hypothesis is bolstered by two distinct studies. First, a study that used *Rag2*-reporter mice to visualize TCR rearrangements in T cell maturation demonstrated an age-related decline in the production of Tregs in the thymus. Furthermore, this decrease occurs at a faster rate than that of non-Tregs, which may encompass pathogenic autoreactive T cells [23]. In addition, investigations that have examined the development of transplanted antigen-specific Tregs in peripheral tissues have revealed that this process wanes with age. Specifically, male-specific Tregs have been generated by transplanting male mouse skin into male antigen-specific TCR transgenic *Rag2*^−/−^ female mice [24]. Because antigen specificity is important in the immunosuppressive function of Tregs, a decrease in the diversity of antigen specificity of Tregs may result in their reduced ability to suppress autoimmune reactions in aged individuals [10, 21, 25]. Consistent with these suggestions, autoantibody production is more likely to appear with aging in general [2, 3]. In terms of cutaneous autoimmune diseases, pemphigus vulgaris (PV) and bullous pemphigoid usually occur in middle-aged and older adults, with peaks of occurrence in those in their 40–60 s and older than 60, respectively [26–28].

PV is a disease in which autoantibodies against desmoglein 3 (Dsg3), an adhesive protein of keratinocytes, appear and disturb epidermal cell adhesion, resulting in blister formation [29]. We previously generated a Dsg-3-specific TCR transgenic mouse, Dsg3H1, which was used to demonstrate that CD4^+^ helper T cells are crucial for autoantibody production [30, 31] and to examine the pathomechanism of anti-Dsg3 autoimmunity. Upon Dsg3 antigen presentation in the thymus, Dsg3H1 T cells undergo central immune tolerance. However, in the *Rag2*-intact condition, endogenous TCR expression overcomes the exclusion, leading to only partial deletion of Dsg3H1 T cells and to their development and distribution in the periphery. These Dsg3H1 T cells can cause skin inflammation, by exhibiting cytotoxic activity against Dsg3-expressing keratinocytes and promoting anti-Dsg3 antibody production, which results in blister formation [32]. When Dsg3H1 mice are bred with *Rag2*^−/−^ mice to eliminate the influence of endogenous TCR, Dsg3H1-*Rag2*^−/−^, T cells are entirely deleted in the Dsg3-expressing thymus [12]. Therefore, Dsg3H1 T cells are useful for examining immunological tolerance against Dsg3 and are deleted in peripheral tissues after Dsg3 antigen recognition in skin-draining lymph nodes (SLNs) in 8-week-old mice [12]. Using this model, we examined changes in peripheral immune tolerance with age. Dsg3H1-*Rag2*^−/−^ T cells exhibited heightened markers indicating activation and evaded elimination in aged mice. These findings are consistent with mechanisms in the early pathogenesis of autoimmune disorders in elderly individuals.

## Results

**Fig. 1:**
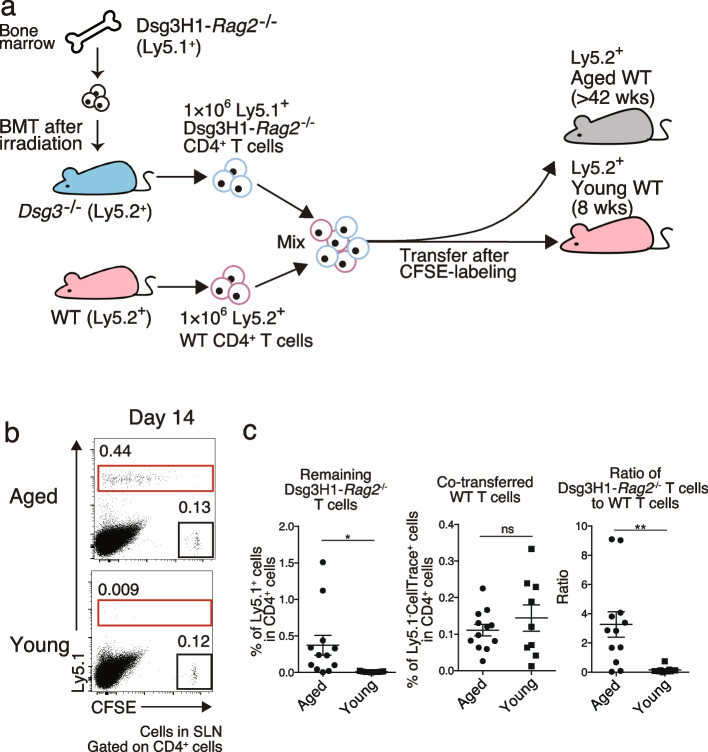
Deletion of Dsg3H1-*Rag2*^−/−^ T cells was disrupted in aged mice. **a** Outline of the adoptive transfer of Dsg3H1-*Rag2*^−/−^ T cells and WT CD4^+^ T cells to aged or young mice. **b**, **c** Flow cytometric (FCM) plots and quantitative summaries of aged and young mice after the transfer of 1 × 10^6^ CFSE-labeled Ly5.1^+^ Dsg3H1-*Rag2*^−/−^ T cells and 1 × 10^6^ Ly5.2^+^ WT T cells as an internal control (gated on CD4^+^). Proliferated CFSE^low^Ly5.1^+^Dsg3H1-*Rag2*^−/−^ T cells and CFSE^+^Ly5.1^−^ co-transferred WT T cells were gated (red and black squares, respectively). Remaining Dsg3H1-*Rag2*^−/−^ T cell ratio was calculated as the proportion of CFSE^low^Ly5.1^+^Dsg3H1-*Rag2*^−/−^ T cells (red squares) divided by that of CFSE^+^Ly5.1.^−^ WT T cells (black squares). The gating strategy was the same as that shown in Fig. S1b. Data are shown as the mean ± SEM. *P < 0.05, **P < 0.01 (unpaired *t*-test). Data were pooled from three independent experiments (*n* = 3–5 mice per group)

We prepared Dsg3H1-*Rag2*^*−/−*^ T cells that had not previously encountered Dsg3 antigen by isolating them from *Dsg3*^*−/−*^ mice. These cells were isolated based on their specific expression of V β6 and were then adoptively transferred into wild-type (WT) mice to examine the behavior of Dsg3-specific T cells capable of recognizing Dsg3 antigen in peripheral tissues (Fig. 1a and Fig. S1a). When WT CD4^+^ T cells identifiable by the congenic markers Ly5.1 or 2 are transferred, they can be used as controls. In addition, CellTrace CFSE labeling prior to transfer can be used to distinguish recipient CD4^+^ T cells from co-transfected WT CD4^+^ T cells and to observe Dsg3H1-*Rag2*^−/−^ T cell proliferation. As previously reported [12], Dsg3H1-*Rag2*^−/−^ T cells proliferated in WT mice at 3 days post-transfer, but no proliferation occurred when the cells were transferred into *Dsg3*^−/−^ mice and observed using the same CFSE intensity as applied to the co-transferred WT T cells. These results suggest that the proliferation of Dsg3H1-*Rag2*^−/−^ T cells is Dsg3 antigen-responsive (Figure S1a and b). We also reported the disappearance of transferred Dsg3-specific T cells within 14 days even after their proliferation in young, (8-week-old) WT mice via peripheral tolerance [12]. In this study, to examine whether this peripheral tolerance mechanism is altered with age, Dsg3-specific T cells were transferred into aged (> 42-week-old) mice. For quantitative comparisons, the same numbers of Dsg3H1-*Rag2*^*−/−*^ and WT CD4^+^ T cells were co-transferred into young and aged mice. The ratio of Dsg3H1-*Rag2*^*−/−*^ T cells to co-transferred WT CD4^+^ T cells was compared between young and aged mice on day 14 after transfer. Significantly more Dsg3H1-*Rag2*^*−/−*^ T cells remained after proliferation in SLNs of aged mice, while Dsg3H1-*Rag2*^*−/−*^ T cells were deleted in young mice (Fig. 1b and c). Despite the absence of obvious clinical or histopathological dermatitis in the aged mice, these results suggest that peripheral tolerance against Dsg3-specific T cells is at least partially disrupted in aged mice.

**Fig. 2:**
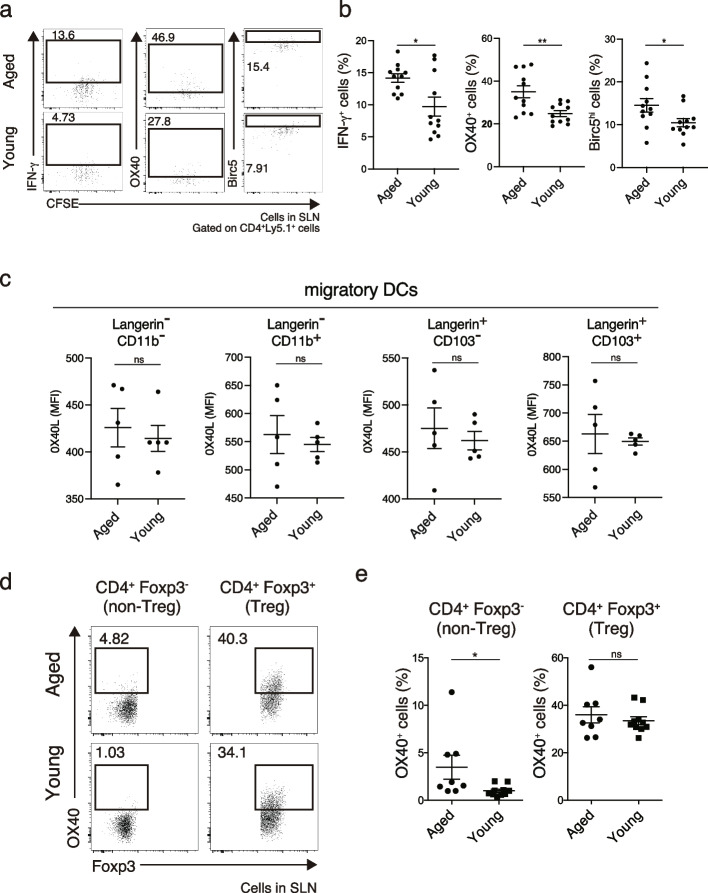
Dsg3H1-*Rag2*^−/−^ T cells were more activated and showed higher Birc5 expression in aged mice. **a**, **b** FCM plots and quantitative summaries of the expression of the indicated factors in Dsg3H1-*Rag2*^−/−^ T cells in SLNs on day 3 after transfer into aged and young mice (gated on Ly5.1^+^CD4^+^ cells; The gating strategy is shown in Fig. S2a). **c** Quantitative summaries of the OX40L expression levels in four subsets of migratory DCs in SLN of aged and young mice: Langerin^−^CD11b^−^, Langerin^−^CD11b^+^, Langerin^+^CD103^−^, and Langerin^+^CD103^+^ cells. The gating strategy is shown in Fig. S2b. **d**, **e** FCM plots and quantitative summaries of OX40 expression in CD4^+^Foxp3^−^ non-Tregs and CD4^+^Foxp3^+^ Tregs in SLNs in aged and young mice without transfer of Dsg3H1-*Rag2*^−/−^ T cells (The gating strategy is shown in Fig. S2c). Data are shown as the mean ± SEM. ns, not significant, *P < 0.05, **P < 0.01 (unpaired *t*-test between groups). Data were pooled from three independent experiments (*n* = 3–5 mice per group) for **a**, **b**. Data are from two independent experiments (*n* = 3–5 mice per group) for **c**-**e**

Next, further experiments were performed to investigate the mechanism underlying the disappearance of these T cells. A previous study reported suppressed pathogenicity of cells during the disappearance of Dsg3H1-*Rag2*^*−/−*^ T cells, by peripheral immune tolerance, consisting of reduced production of the inflammatory cytokine IFN-γ after antigen recognition [12]. Then IFN-γ production was compared in Dsg3H1-*Rag2*^*−/−*^ T cells, which mainly proliferate, on day 3 after transfer between young and aged mice. The production levels were higher in aged mice than in young mice (Fig. 2a and b, and Fig. S2a). This result suggests that the suppressed pathogenicity observed in Dsg3-specific T cells, which are destined to be deleted by peripheral tolerance, became less apparent in aged mice.

Both OX40 and Birc5, a downstream molecule of OX40 signaling, were upregulated to a greater extent in aged than in young mice (Fig. 2a and b). A critical role for the restriction of OX40 signaling in Dsg3H1-*Rag2*^*−/−*^ T cell disappearance was previously reported [12]. To investigate the possible age-related elevated expression of OX40L on migrating dendritic cells in skin lymph nodes that can present Dsg3 antigen to Dsg3H1-*Rag2*^*−/−*^ T cells, OX40L expression on four subsets of migratory DCs (Langerin^*−*^CD11b^*−*^, Langerin^*−*^CD11b^+^, Langerin^+^CD103^*−*^, and Langerin^+^CD11b^+^ DCs) was compared in aged and young mice, but there were no significant differences(Fig. 2c and Fig. S2b). Next, as OX40 signaling in Dsg3H1-*Rag2*^*−/−*^ T cells is attenuated in a Treg OX40-dependent manner [12], we compared the expression level of OX40 on Tregs in the SLNs of young and aged mice. However, there was no apparent difference between different age groups, while the level of OX40 expression in non-Treg CD4^+^ T cells was higher in aged mice (Fig. 2d and e, and Fig. S2c). Although Treg OX40-dependent regulation of OX40 signaling in disappearing T cells was not observed in aged mice, these observations suggested that OX40 and Birc5 upregulation during proliferation contributes to the mechanism of Dsg3H1 T cell escape from peripheral disappearance in aged mice.

## Discussion

In autoreactive Dsg3H1-*Rag2*^*−/−*^ T cells eliminated by peripheral immune tolerance in young mice OX40 and Birc5 expression were upregulated and escaped elimination in aged mice. OX40 on Tregs was shown to restrain OX40 signaling in Dsg3H1-*Rag2*^*−/−*^ T cell elimination as part of the mechanism of peripheral tolerance [12]. Thus, the age-dependent increase of OX40L on migrating DCs and the decrease of Treg OX40 may explain the upregulation of OX40 and Birc5 with age. However, the expression of OX40L on migrating DCs nor Treg OX40 did not change significantly with age. Alternatively, non-Treg T cells, including Dsg3H1-*Rag2*^*−/−*^ T cells, may be more likely to express OX40 due to general changes in the immunological environment with aging, as can be inferred by the increase in OX40 expression on non-Treg CD4^+^ T cells in aged mice (Fig. 2d and e). The reason for this increase is unclear, but it may be related to changes in the expression of cytokines, such as IL-1α/β, which are inducers of OX40 expression [33]. In fact, increased IL-1β levels has been reported in tissues other than the skin or SLN and IL-1β gene expression was shown to be increased in the CD45^+^ immune cell fraction in pancreatic islets with age [34]. In addition, the levels of plasma IL-1β, produced in the lung and spleen, increased after intraperitoneal injection of lipopolysaccharide in aged mice [35]. Therefore, IL-1β may also be upregulated in lymph nodes, leading to increased OX40 expression in non-Treg cells, including Dsg3H1-*Rag2*^*−/−*^ T cells. However, further studies are required to clarify these issues.

The aging-related changes in OX40 signaling in T cells are complex. A study in an ovalbumin (OVA)-expressing tumor model showed that when OVA-specific CD4 T cells were transferred into tumor-transplanted aged mice (20 months old) and young mice (≤ 6 months old) with OX40 agonist, the OX40 stimulation-induced effector T-cell differentiation of the transferred cells was reduced in aged mice [36]. In our experiment, however, T cells transferred into aged mice showed increased OX40 signaling and activation. This difference may have been due to evaluation under conditions of systemic OX40 stimulation by exogenous reagent in the tumor model, while in our system only endogenous OX40L of the recipient’s cells could stimulate OX40 in transferred T cells. The most important point when considering the effects of systemic OX40 stimulation is that Tregs express OX40 at higher levels than non-Treg CD4^+^ T cells [37]. Systemic OX40 stimulation of aged mice by exogenous reagents may increase the number of Tregs that subsequently suppress effector T cell differentiation, as Treg induction by OX40 stimulation is increased in the presence of high levels of IFN- γ and IL-4 [38], and IFN- γ production by T cells was also reported to increase with age [39].

A notable finding of this study was the quenched pathogenicity of remaining Dsg3H1-*Rag2*^*−/−*^ T cells in the induction of dermatitis. Although the remaining Dsg3H1-*Rag2*^*−/−*^ T cells exerted their pathogenicity in the absence of Tregs or under conditions of systemic OX40 stimulation in young mice [12], in aged mice the remaining Dsg3H1-*Rag2*^*−/−*^ T cells did not always result in the development of the phenotype; rather another mechanism of peripheral tolerance seems to have restrained their pathogenicity instead of inducing cell deletion. The risk of autoantibody detection increases with age, but it does not always cause disease [2, 3]. The increases in the Treg population and IL-10 production with age may contribute to the suppression of antibody-mediated phenotype development [2, 3]. Similar mechanisms may exist to prevent disease-phenotype development by pathogenic autoreactive T cells, although this remains to be explored in further studies.

Our study elucidates one aspect of the fragility of immunological tolerance in the aging population, i.e., pathogenic autoreactive T cells, which were usually deleted in young mice, survived and expressed elevated levels of OX40 and Birc5. As interface dermatitis, an autoimmune inflammatory condition, did not occur despite the survival of these T cells, immunoregulatory mechanisms may have suppressed the development of autoimmune disease, implying peripheral tolerance that remains functional even in the aged population. The survival of autoreactive Dsg3H1-*Rag2*^*−/−*^ T cells may reflect a preliminary step in disease development. Understanding cell survival and the related immunoregulatory mechanisms may contribute to the development of therapies for not only PV but also other autoimmune diseases and to the monitoring of autoimmune disease risk with age.

## Materials and methods

### Mice

C57BL/6J mice (The Jackson Laboratory, Bar Harbor, ME, USA), C57BL/6 *Rag2*^−/−^ mice (Central Institute for Experimental Animals, Kanagawa, Japan), Dsg3H1-tg mice [32], and *Dsg3*^−/−^ mice [40] were bred in the experimental animal care facility of Keio University under specific pathogen-free conditions. For all experiments, 8-week-old and > 42-week-old male and female mice were used.

### Antibodies and flow cytometry

A single-cell suspension of SLNs of mice was stained with antibodies, listed in Table S1, from Thermo Fisher (Cleveland, OH, USA), BioLegend (San Diego, CA, USA), and BD Biosciences (San Jose, CA, USA). We used 7-AAD Viability Staining Solution (BioLegend) or LIVE/DEAD® Fixable Dead Cell Stain (Thermo Fisher) to identify dead cells. Cells were stained for 20 min on ice in staining buffer (phosphate buffered saline containing 2% fetal bovine serum and 5 µg Fcγ R III/II blocking antibody/mL). Flow cytometry was performed on a Canto II and LSRFortessa instrument (BD Biosciences) and data were analyzed using FlowJo (Tree Star, Ashland, OR, USA).

### Intracellular staining

Intracellular staining, for IFN- γ, Birc5 and Foxp3, was performed with antibodies and fixation/permeabilization buffers (BD Biosciences) or Foxp3/transcription factor staining buffer set (Thermo Fisher). For IFN- γ staining, the cells were stained after treatment with phorbol 12-myristate 13 acetate (Sigma-Aldrich, St. Louis, MO, USA), ionomycin calcium salt (Sigma-Aldrich), and Goldiplug (BD Biosciences).

### Bone marrow transplantation

CD4, CD8, and B220-depleted BM cells were prepared using a MACS cell separation system with CD4, CD8, and B220 microbeads (Miltenyi Biotec, Bergisch Gladbach, Germany) from Dsg3H1-*Rag2*^−/−^ mice and 1 × 10^6^ of sorted cells were transferred intravenously into irradiated *Dsg3*^−/−^ mice. The radiation dose was 7.5 Gy for *Dsg3*^−/−^ and WT mice. Recipient mice were used for experiments 2 months later.

### Adoptive transfer

Dsg3H1-*Rag2*^−/−^ T cells (Ly5.1^+^) were prepared from the spleen (Sp), SLNs, and mesenteric lymph nodes of *Dsg3*^−/−^ mice that underwent bone marrow transplantation from Dsg3H1-*Rag2*^−/−^ mice by depletion of B220^+^ and CD8^+^ cells followed by positive selection of Vβ6^+^ cells using magnetic beads (Miltenyi Biotech) according to the manufacturer’s instructions. Control WT CD4^+^ T cells (Ly5.2^+^) were prepared from WT mice by positive selection of CD4^+^ cells using magnetic beads. The isolated Dsg3H1-*Rag2*^−/−^ T cells and WT CD4^+^ T cells were mixed in same number and then labeled with CellTrace CFSE (Thermo Fisher) using an incubation method, where the cells were exposed to 1 uM CFSE for 8 min at room temperature. These labeled cells (1 × 10^6^　cells each) were resuspended in 200 μL phosphate-buffered saline and subsequently adoptively transferred into recipient mice.

## Supplementary Information

**Additional file 1:** **Table S1.** A comprehensive list of antibodies used in the experiments as well as information on the target antigen, clone, type of fluorescence, catalog number, and the respective supplying companies. **Fig. S1.** a,Outline of the adoptive transfer of Dsg3H1-*Rag2*^−/−^ T cells and WT CD4^+^ T cells to *Dsg3*^−/−^ or WT mice. b, FCM plots of *Dsg3*^−/−^and WT mice after the transfer of CFSE-labeled Ly5.1^+^ Dsg3H1-*Rag2*^−/−^ T cells and Ly5.2^+^ WT T cells. The gating strategy used to identify 7AAD^−^ CD4+ cells is illustrated. Ly5.1^+^Dsg3H1-*Rag2*^−/−^ T cells and CFSE^+^Ly5.1^−^ co-transferred WT T cells were gated (red and black squares, respectively). Proliferation indicated by CFSE intensity reduction of Ly5.1^+^Dsg3H1-*Rag2*^−/−^ T cells are observed only in WT mice. **Fig. S2.** a, FCM plots demonstrate the gating strategy employed to identify proliferating Ly5.1^+^ Dsg3H1-*Rag2*^*−/−*^ Tcells in SLN. Cutoff values for the expression of IFN-γ, Birc5, and OX40 in Dsg3H1-*Rag2*^*−/−*^ T cells were based on their expression levels in recipient T cells. Fluorescence minus one (FMO) controls of CD4, Ly5.1,CFSE, IFN-γ, Birc5, and OX40 are also shown. b, FCM plots show the gating strategy employed to identify four subsets of migrating dendritic cells in SLN. FMO controls of CD45, CD11c, IA/IE, CD11b, Langerin and CD103 are also shown. c, FCM plots demonstrate the gating strategy employed to identify Tregs in SLN. FMO controls of Foxp3 and OX40 are also shown.

## Data Availability

The datasets used and/or analyzed during the current study are available from the corresponding author on reasonable request.

## References

[1] Gascoigne NR, Rybakin V, Acuto O, Brzostek J (2016). TCR signal strength and T Cell development. Annu Rev Cell Dev Biol.

[2] Vadasz Z, Haj T, Kessel A, Toubi E (2013). Age-related autoimmunity. BMC Med.

[3] Andersen-Ranberg K (2004). M HO-M, Wiik A, Jeune B, Hegedus L: High prevalence of autoantibodies among Danish centenarians. Clin Exp Immunol.

[4] Derbinski J, Schulte A, Kyewski B, Klein L (2001). Promiscuous gene expression in medullary thymic epithelial cells mirrors the peripheral self. Nat Immunol.

[5] Anderson MS, Venanzi ES, Klein L, Chen Z, Berzins SP, Turley SJ, von Boehmer H, Bronson R, Dierich A, Benoist C (2002). Projection of an immunological self shadow within the thymus by the aire protein. Science.

[6] Takaba H, Morishita Y, Tomofuji Y, Danks L, Nitta T, Komatsu N, Kodama T, Takayanagi H (2015). Fezf2 Orchestrates a Thymic Program of Self-Antigen Expression for Immune Tolerance. Cell.

[7] Klein L, Robey EA, Hsieh CS (2019). Central CD4(+) T cell tolerance: deletion versus regulatory T cell differentiation. Nat Rev Immunol.

[8] Sojka DK, Huang YH, Fowell DJ (2008). Mechanisms of regulatory T-cell suppression - a diverse arsenal for a moving target. Immunology.

[9] Schmidt A, Oberle N, Krammer PH (2012). Molecular mechanisms of treg-mediated T cell suppression. Front Immunol.

[10] Shevyrev D, Tereshchenko V (2019). Treg heterogeneity, function, and homeostasis. Front Immunol.

[11] Kretschmer K, Apostolou I, Hawiger D, Khazaie K, Nussenzweig MC, von Boehmer H (2005). Inducing and expanding regulatory T cell populations by foreign antigen. Nat Immunol.

[12] Iriki H, Takahashi H, Wada N, Nomura H, Mukai M, Kamata A, Ito H, Yamagami J, Matsui T, Kurebayashi Y, et al. Peripheral tolerance by Treg via constraining OX40 signal in autoreactive T cells against desmoglein 3, a target antigen in pemphigus. Proc Natl Acad Sci U S A. 2021;118(49):e2026763118. 10.1073/pnas.2026763118 PMC8670434 34848535

[13] Ritprajak P, Kaewraemruaen C, Hirankarn N. Current Paradigms of Tolerogenic Dendritic Cells and Clinical Implications for Systemic Lupus Erythematosus. Cells. 2019;8(10):1291. 10.3390/cells8101291 PMC6830089 31640263

[14] Castenmiller C, Keumatio-Doungtsop BC, van Ree R, de Jong EC, van Kooyk Y (2021). Tolerogenic immunotherapy: targeting DC surface receptors to induce antigen-specific tolerance. Front Immunol.

[15] Iberg CA, Jones A, Hawiger D (2017). Dendritic cells As inducers of peripheral tolerance. Trends Immunol.

[16] Sakaguchi S, Wing K, Yamaguchi T (2009). Dynamics of peripheral tolerance and immune regulation mediated by Treg. Eur J Immunol.

[17] Qi Q, Liu Y, Cheng Y, Glanville J, Zhang D, Lee JY, Olshen RA, Weyand CM, Boyd SD, Goronzy JJ (2014). Diversity and clonal selection in the human T-cell repertoire. Proc Natl Acad Sci U S A.

[18] Goronzy JJ, Weyand CM (2017). Successful and maladaptive T cell aging. Immunity.

[19] Uciechowski P, Kahmann L, Plumakers B, Malavolta M, Mocchegiani E, Dedoussis G, Herbein G, Jajte J, Fulop T, Rink L (2008). TH1 and TH2 cell polarization increases with aging and is modulated by zinc supplementation. Exp Gerontol.

[20] Garg SK, Delaney C, Toubai T, Ghosh A, Reddy P, Banerjee R, Yung R (2014). Aging is associated with increased regulatory T-cell function. Aging Cell.

[21] Rocamora-Reverte L, Melzer FL, Wurzner R, Weinberger B (2020). The complex role of regulatory T cells in immunity and aging. Front Immunol.

[22] Chougnet CA, Tripathi P, Lages CS, Raynor J, Sholl A, Fink P, Plas DR, Hildeman DA (2011). A major role for Bim in regulatory T cell homeostasis. J Immunol.

[23] Thiault N, Darrigues J, Adoue V, Gros M, Binet B, Perals C, Leobon B, Fazilleau N, Joffre OP, Robey EA (2015). Peripheral regulatory T lymphocytes recirculating to the thymus suppress the development of their precursors. Nat Immunol.

[24] Carpentier M, Chappert P, Kuhn C, Lalfer M, Flament H, Burlen-Defranoux O, Lantz O, Bandeira A, Malissen B, Davoust J (2013). Extrathymic induction of Foxp3(+) regulatory T cells declines with age in a T-cell intrinsic manner. Eur J Immunol.

[25] Hoeppli RE, MacDonald KG, Levings MK, Cook L (2016). How antigen specificity directs regulatory T-cell function: self, foreign and engineered specificity. HLA.

[26] Kridin K, Ludwig RJ (2018). The growing incidence of bullous pemphigoid: overview and potential explanations. Front Med (Lausanne).

[27] Persson MSM, Harman KE, Vinogradova Y, Langan SM, Hippisley-Cox J, Thomas KS, Gran S (2021). Incidence, prevalence and mortality of bullous pemphigoid in England 1998–2017: a population-based cohort study. Br J Dermatol.

[28] Meyer N, Misery L (2010). Geoepidemiologic considerations of auto-immune pemphigus. Autoimmun Rev.

[29] Amagai M, Klaus-Kovtun V, Stanley JR (1991). Autoantibodies against a novel epithelial cadherin in pemphigus vulgaris, a disease of cell adhesion. Cell.

[30] Zhang XM, Liu CY, Shao ZH (2020). Advances in the role of helper T cells in autoimmune diseases. Chin Med J (Engl).

[31] Crotty S (2015). A brief history of T cell help to B cells. Nat Rev Immunol.

[32] Takahashi H, Kouno M, Nagao K, Wada N, Hata T, Nishimoto S, Iwakura Y, Yoshimura A, Yamada T, Kuwana M (2011). Desmoglein 3-specific CD4+ T cells induce pemphigus vulgaris and interface dermatitis in mice. J Clin Invest.

[33] Nakae S, Asano M, Horai R, Sakaguchi N, Iwakura Y (2001). IL-1 enhances T cell-dependent antibody production through induction of CD40 ligand and OX40 on T cells. J Immunol.

[34] Boni-Schnetzler M, Mereau H, Rachid L, Wiedemann SJ, Schulze F, Trimigliozzi K, Meier DT, Donath MY (2021). IL-1beta promotes the age-associated decline of beta cell function. iScience.

[35] Starr ME, Saito M, Evers BM, Saito H (2015). Age-Associated Increase in Cytokine Production During Systemic Inflammation-II: The Role of IL-1beta in Age-Dependent IL-6 Upregulation in Adipose Tissue. J Gerontol A Biol Sci Med Sci.

[36] Ruby CE, Weinberg AD (2009). OX40-enhanced tumor rejection and effector T cell differentiation decreases with age. J Immunol.

[37] Remedios KA, Zirak B, Sandoval PM, Lowe MM, Boda D, Henley E, Bhattrai S, Scharschmidt TC, Liao W, Naik HB, et al. The TNFRSF members CD27 and OX40 coordinately limit TH17 differentiation in regulatory T cells. Sci Immunol. 2018;3(30):eaau2042. 10.1126/sciimmunol.aau2042 30578350

[38] Ruby CE, Yates MA, Hirschhorn-Cymerman D, Chlebeck P, Wolchok JD, Houghton AN, Offner H, Weinberg AD (2009). Cutting Edge: OX40 agonists can drive regulatory T cell expansion if the cytokine milieu is right. J Immunol.

[39] Sakata-Kaneko S, Wakatsuki Y, Matsunaga Y, Usui T, Kita T (2000). Altered Th1/Th2 commitment in human CD4+ T cells with ageing. Clin Exp Immunol.

[40] Hata T, Nishifuji K, Shimoda K, Sasaki T, Yamada T, Nishikawa T, Koyasu S, Amagai M (2011). Transgenic rescue of desmoglein 3 null mice with desmoglein 1 to develop a syngeneic mouse model for pemphigus vulgaris. J Dermatol Sci.

