# Supplementary material for: Attenuation of OX40 signaling suppression by age disrupts peripheral deletion of CD4+ T cells specific for the epidermal autoantigen desmoglein 3
Source: Immun Ageing. 2023 Jun 12;20:26. doi: 10.1186/s12979-023-00353-9 (PMC10259059; doi:10.1186/s12979-023-00353-9)
Supplement: Supplementary file 1 — Additional file 1: Table S1. A comprehensive list of antibodies used in the experiments as well as information on the target antigen, clone, type of fluorescence, catalog number, and the respective supplying companies. Fig. S1. a,Outline of the adoptive transfer of Dsg3H1-Rag2−/− T cells and WT CD4+ T cells to Dsg3−/− or WT mice. b, FCM plots of Dsg3−/−and WT mice after the transfer of CFSE-labeled Ly5.1+ Dsg3H1-Rag2−/− T cells and Ly5.2+ WT T cells. The gating strategy used to identify 7AAD− CD4+ cells is illustrated. Ly5.1+Dsg3H1-Rag2−/− T cells and CFSE+Ly5.1− co-transferred WT T cells were gated (red and black squares, respectively). Proliferation indicated by CFSE intensity reduction of Ly5.1+Dsg3H1-Rag2−/− T cells are observed only in WT mice. Fig. S2. a, FCM plots demonstrate the gating strategy employed to identify proliferating Ly5.1+ Dsg3H1-Rag2−/− Tcells in SLN. Cutoff values for the expression of IFN-γ, Birc5, and OX40 in Dsg3H1-Rag2−/− T cells were based on their expression levels in recipient T cells. Fluorescence minus one (FMO) controls of CD4, Ly5.1,CFSE, IFN-γ, Birc5, and OX40 are also shown. b, FCM plots show the gating strategy employed to identify four subsets of migrating dendritic cells in SLN. FMO controls of CD45, CD11c, IA/IE, CD11b, Langerin and CD103 are also shown. c, FCM plots demonstrate the gating strategy employed to identify Tregs in SLN. FMO controls of Foxp3 and OX40 are also shown. [file 12979_2023_353_MOESM1_ESM.pdf]

Table S1

Target	Clone	Fluorescence	Catalog #	Company
IFN-γ	XMG1.2	PE	505808	Biolegend
IFN-γ	XMG1.2	PerCP/Cy5.5	505822	Biolegend
Foxp3	FJK-16s	PE/Cy7	25-5773-82	Thermo Fisher
Langerin	4C7	APC	144206	Biolegend
CD4	RM4-5	APC/Cy7	100526	Biolegend
CD4	RM4-5	PE/Cy7	100528	Biolegend
CD4	RM4-5	BUV395	740208	BD bioscience
CD11c	N418	APC/Cy7	117324	Biolegend
CD11b	M1/70	BUV395	565976	BD bioscience
Ly5.1	A20	BUV395	565212	BD bioscience
Ly5.1	A20	BUV737	612811	BD bioscience
Ly5.1	A20	BV421	110732	Biolegend
OX40	OX-86	PE	119410	Biolegend
OX40L	RM134L	PE	108806	Biolegend
IA/IE	M5/114.15.2	FITC	107606	Biolegend
CD103	2E7	BV421	121422	Biolegend
Birc5 / Survivin	Polyclonal	Unconjugated	NB500-201	Novus bioloical
Rabbit IgG	Polyclonal	APC	A-10931	Thermo Fisher

Fig.S1

a

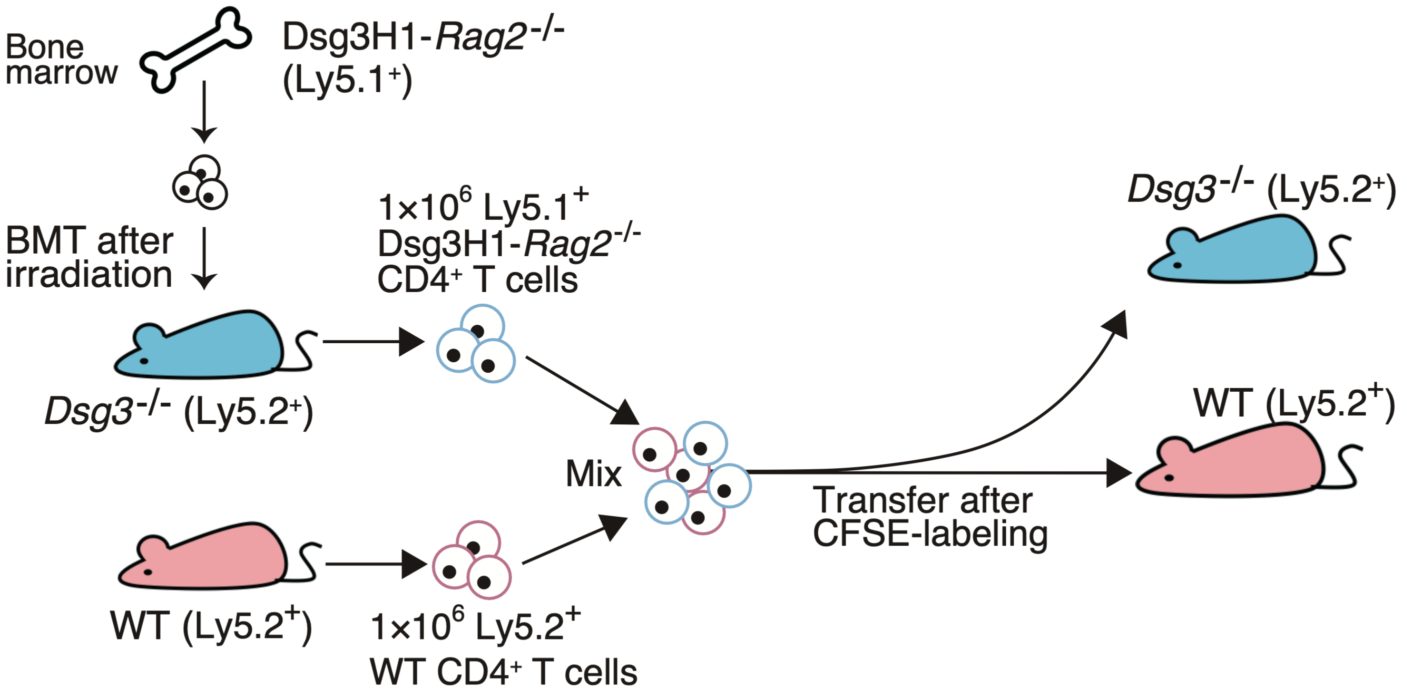

b

Day 3 after transfer

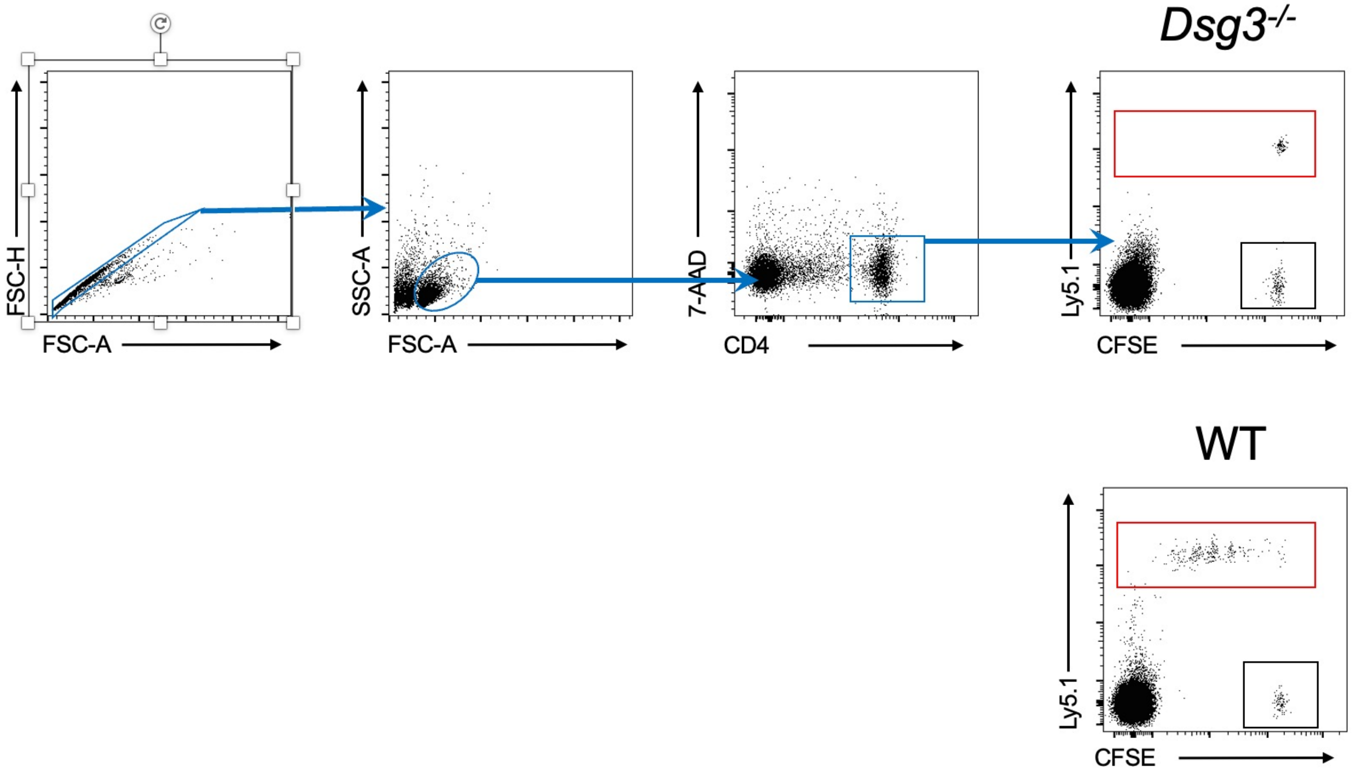

Fig.S2

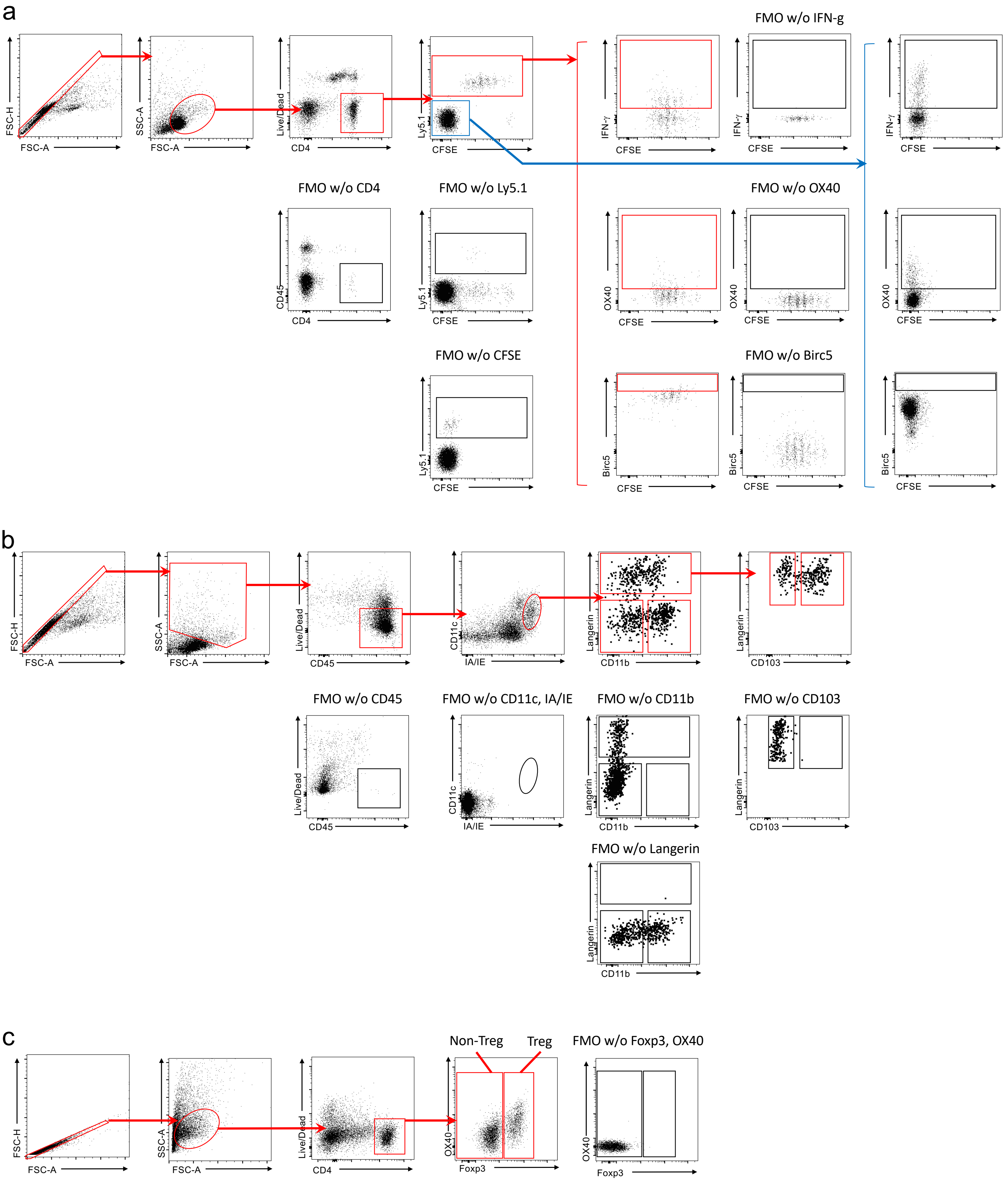
a
FSC-H
FSC-A
SSC-A
FSC-A
Live/Dead
CD4
Ly5.1
CFSE
IFN-γ
CFSE
FMO w/o IFN-g
IFN-γ
CFSE
IFN-γ
CFSE
FMO w/o CD4
CD45
CD4
FMO w/o Ly5.1
Ly5.1
CFSE
FMO w/o OX40
OX40
CFSE
OX40
CFSE
OX40
CFSE
FMO w/o CFSE
Ly5.1
CFSE
FMO w/o Birc5
Birc5
CFSE
Birc5
CFSE
Birc5
CFSE
b
FSC-H
FSC-A
SSC-A
FSC-A
Live/Dead
CD45
CD11c
IA/IE
Langerin
CD11b
Langerin
CD103
FMO w/o CD45
Live/Dead
CD45
FMO w/o CD11c, IA/IE
CD11c
IA/IE
FMO w/o CD11b
Langerin
CD11b
FMO w/o CD103
Langerin
CD103
FMO w/o Langerin
Langerin
CD11b
c
FSC-H
FSC-A
SSC-A
FSC-A
Live/Dead
CD4
Non-Treg
Treg
OX40
Foxp3
FMO w/o Foxp3, OX40
OX40
Foxp3
